# Antimicrobial Resistance of *Enterococcus* Isolates from Poultry Farms in the Republic of Serbia (Autonomous Province of Vojvodina)

**DOI:** 10.3390/microorganisms12071483

**Published:** 2024-07-20

**Authors:** Maja Velhner, Bojana Prunić, Nevenka Aleksić, Dalibor Todorović, Slobodan Knežević, Dragana Ljubojević Pelić

**Affiliations:** 1 Scientific Veterinary Institute “Novi Sad”, 21000 Novi Sad, Serbia; 2 Faculty of Veterinary Medicine, University of Belgrade, 11000 Belgrade, Serbia

**Keywords:** antimicrobial susceptibility, MALDI-TOF mass spectrometry, *Enterococcus* spp. identification, public health, control measures

## Abstract

*Enterococcus* species are significant intestinal commensals of animals, including poultry. However, they have emerged as important opportunistic infective agents in both veterinary and human medicine as well as major nosocomial pathogens, owing to their increasing antimicrobial resistance. This research aimed to investigate the prevalence and antimicrobial resistance patterns of *Enterococcus* spp. isolated from poultry farms in the north of Serbia. A total of 40 samples of overshoes or feces were collected from 40 poultry farms and analyzed for the presence of *Enterococcus* spp. using PCR or MALDI-TOF mass spectrometry for their identification. The number of isolates was 40 and included 11 isolates from laying hens, 2 isolates from turkeys, 3 from broiler breeders, and 24 from broilers. The Kirby–Bauer disk diffusion method was used to test for antibiotic susceptibility in accordance with the Clinical and Laboratory Standards Institute and EUCAST guidelines. The results showed that *Enterococcus faecalis* was isolated from 37.5% farms, and *E. faecium* from 42.5%. *E. hirae* was identified in 15% of poultry establishments, and *E. durans* and *E. thialandicus* on 2.5%. Notably, resistance to erythromycin, streptomycin, fluoroquinolones, and tetracyclines among the frequently used antibiotics was found. Furthermore, 35% of the isolates had multidrug resistance (MDR). In order to prevent the spread of antibiotic resistance in chicken farming and protect the health of the public and animals alike, our findings highlight the critical need for improved surveillance and control measures. To effectively establish a containment strategy for *Enterococcus* spp. isolated from poultry farms, more research into the processes behind their antibiotic resistance is required.

## 1. Introduction

*Enterococcus* spp. are among the most important nosocomial pathogens worldwide [1,2,3]. As harmless commensals, they colonize the intestines of humans and animals, while poultry, in particular, are their permanent reservoirs [4]. Humans may be infected with pathogenic enterococci, but the route of infection is not always known. It is considered that the most common source of pathogenic *Enterococcus* spp. is food of animal origin, and people can contract the infection when handling poultry meat in slaughterhouses or food factories [5].

Antimicrobial resistance (AMR) is a global public health concern that threatens the effectiveness of antimicrobials used to treat bacterial infections [6]. Recently, it has been determined that the most significant poultry bacteria resistant to antibiotics in the European Union are *Escherichia coli*, *Enterococcus faecalis*, and *Enterococcus cecorum* [7]. Therefore, in many countries, the antimicrobial resistance monitoring of *Enterococcus* spp. has been initiated, although currently it is not mandatory in the EU [8]. There are several reasons why these bacteria require special attention. Namely, *Enterococcus* spp. survive in different environments and hosts, owing to their specific resistance phenotypes, virulence and good adaptability, withstanding high temperatures, salinity, and acidic conditions [9]. Enterococci are easily transmitted in chicken flocks and survive well in the environment [10]. The isolates can become pathogenic for chickens and their embryos [8]. Furthermore, in poultry farming, where antimicrobials are commonly used for disease prevention but also for growth promotion [11], *Enterococcus* spp. have garnered attention as potential reservoirs and vectors for the dissemination of antimicrobial resistance genes [12]. These are perfectly capable of exchanging resistance and virulence genes between themselves and other bacteria [13].

Certain strains, such as *Enterococcus cecorum*, which are pathogenic for poultry [14], do not typically colonize human guts and seldom cause bacteriemia [15]. However, *E. cecorum* was isolated from a patient’s pleural fluid and the intestines were thought to be the portals of entry [16]. 

*E. faecalis* is an opportunistic pathogen that may cause outbreaks in poultry flocks if they are infected concomitantly with other microorganisms or suffer from immunosuppression. Some clones such as sequence type (ST) 82 may cause amyloid arthropathy in broiler breeders and are considered pathogenic [8,17]. 

The Autonomous Province of Vojvodina, situated in the north of Serbia, is a significant area for poultry production in the region. Alongside the intensification of poultry farming and the widespread use of antimicrobials, concerns have been raised regarding the emergence and spread of AMR among *Enterococcus* spp. isolates in poultry production. Understanding the prevalence and patterns of antimicrobial resistance in *Enterococcus* spp. from poultry farms in Vojvodina is crucial for developing strategies aimed to mitigate the risk of AMR transmission within the food chain and to human populations. To the best of our knowledge, based on literature search, this study presents the first investigation into *Enterococcus* spp. resistance on poultry farms in Serbia. Moreover, there is a lack of data pertaining to the occurrence and antimicrobial resistance of *Enterococcus* spp. in surrounding countries. 

In this work, the presence of *Enterococcus* spp. in the environment of the poultry farms in two districts in Vojvodina was investigated. The research goals were to reveal antimicrobial resistance phenotype and estimate the risks of antibiotic overuse in primary production farms in Serbia. Furthermore, the data about the levels of antimicrobial resistance in *Enterococcus* spp. will enable the comparison of isolates from different laboratories and, ultimately, from different countries. The results may help enhance therapeutic efficacy, monitor clonal dissemination, and determine the possible role of food contamination with *Enterococcus* spp. in public health. In addition, the acquired knowledge is essential for the development of targeted interventions and programs to preserve the effectiveness of antimicrobial therapy and safeguard public health.

## 2. Materials and Methods

### 2.1. Poultry Farms

**Figure 1:**
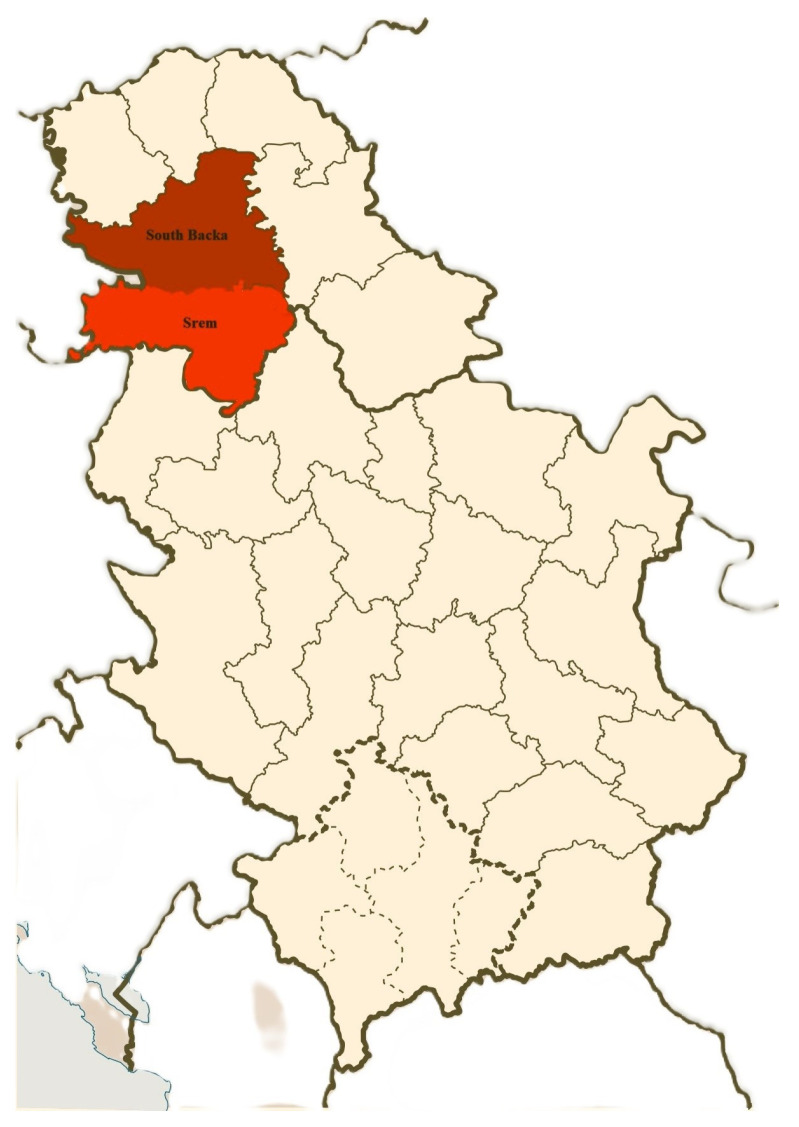
The map of the examined epizootiological areas (South Backa and Srem districts).

The poultry farms investigated in this research are situated in the northern part of Serbia, the Autonomous Province of Vojvodina. The epizootiological area in our study encompasses two districts: South Backa and Srem (Figure 1). We made sure to include all representative farms from these districts to ensure comprehensive coverage of the region. A total of 40 poultry farms, including 3 broiler breeder farms, 11 layer chicken farms, 24 broiler farms, and 2 turkey farms, were examined for the presence of *Enterococcus* spp. in the farm environment. 

Regarding the type of feed, all farms included in our study used commercial feed formulated according to the age of the birds. This ensured that the nutritional requirements of the birds were met throughout their growth stages. All birds were vaccinated according to specific vaccination programs tailored to their needs. These programs are designed to protect against common diseases and ensure the health and well-being of the poultry. The exact vaccination schedule for each flock and category of poultry is created individually; some of the diseases included in the vaccination program are infectious bronchitis, Gumboro disease, and Newcastle disease. To the best of our knowledge, no growth promoters were administered on any of the farms surveyed. This aligns with current best practices and regulatory standards aimed at promoting safe and sustainable poultry production. All of the farms included in our study are commercial closed poultry farms that adhere to high biosecurity measures. As part of these measures, there are no other animals, such as hogs or cattle, present on or near these farms. This strict biosecurity protocol is implemented to ensure the health and safety of the poultry. All farms are single-age farms.

The samples were overshoes or feces collected during the compulsory monitoring of *Salmonella* spp. according to the *Rulebook on determining measures for early detection, diagnosis, prevention of spread, control, and eradication of poultry infection with certain Salmonella serovars* [18], which is almost identical to the respective EU regulations. Also, we used the accompanying *Salmonella* spp. sampling manual for poultry flocks issued by the Serbian Veterinary Directorate (https://www.vet.minpolj.gov.rs/aktuelnosti/samoneloza%20zivine/98.pdf, accessed on 15 May 2024). 

The number of samples collected and analyzed from each farm was one composite environmental sample per farm (feces or overshoes, as described in the manual). This sampling process was conducted strictly in accordance with established regulations to ensure consistency and representativeness. 

All samples were collected following standardized procedures to ensure their integrity and reliability. Once collected, the samples were immediately transported to the laboratory under controlled conditions to maintain their quality. All samples were processed the same day for bacteriological analysis according to established protocols. 

### 2.2. Isolation and Identification of Enterococcus spp.

The total number of isolates was 40 and included 11 isolates from laying hens, 2 isolates from turkeys, 3 from broiler breeders, and 24 from broilers. *Enterococcus* spp. were isolated after inoculation in buffer peptone water upon incubation for 24 h at 37 °C. the next day, 100 μL of inoculated peptone was transferred to Slanetz Bartley medium (Oxoid CM0377B) to obtain single colonies. After incubation for 24–48 h, a single colony of *Enterococcus* sp. was transferred to Bile Aesculin agar (Oxoid CM0888) to obtain pure culture. In each case, the growth was visible after 24 h of incubation at 37 °C. 

The identification of *Enterococcus* spp. was achieved either by PCR or MALDI-TOF. PCR assays were run with the commercial kit BioLabs (New England BioLabs) in accordance with the manufacturer’s instructions using primers recommended by the Danish Technical University (DTU). The sequences of the primers were as follows: *E. faecalis* E1-5′ATCAAGTACAGTTAGTCTT and E2-5′-ACGATTCAAAGCTAACTG-3′, *E. faecium* F1-5′-GCAAGGCTTCTTAGAGA-3′ and F2-5′-CATCGTGTAAGCTAACTTC-3′. The cycles for the PCR were as follows: 94 °C for 2 min, and then the master cycler was programmed for 30 cycles at 94 °C for 1 min., 54 °C for 1 min., and 78 °C for 1 min., with the last cycle being 78 °C 10 min [19]. The PCR reaction yielded products of 941 bp for *E. faecalis* and 550 bp for *E. faecium*. MALDI-TOF mass spectrometry was used for the identification of all isolates that were not possible to identify using PCR.

### 2.3. Antimicrobial Susceptibility Testing

Antibiotic susceptibility was established by the disk diffusion method. The results were interpreted in compliance with protocols provided by the Clinical and Laboratory Standards Institute CLSI M100 [20] and EUCAST document [21]. The following disks (BioRad, Mames-la-Coquette, France) were used: ampicillin (AMP) 10 μg, ciprofloxacin (CIP) 5 μg, erythromycin (ERY)15 μg, chloramphenicol (CHL) 30 μg, tetracycline (TET) 30 μg, gentamicin (GMN) 30 μg, nitrofurantoin (FTN) 300 μg, fosfomycin (FOS) 200 μg, quinupristin–dalfopristin (QDF) 15 μg, linezolid (LZD) 30 μg, vancomycin (VAN) 30 μg, teicoplanin (TEC) 30 μg, tigecycline (TGC) 15 μg, doxycycline (DOX) 30 μg, moxifloxacin (MXF) 5 μg, norfloxacin (NXN) 10 μg, levofloxacin (LVX) 5 μg, and streptomycin (STR300) 300 μg. Quality control was carried out by determining the inhibition zones in *E. coli* ATCC 25922 and *Enterococcus faecalis* ATCC 29212. 

Isolates that demonstrated resistance to three or more antimicrobial drugs from different families were deemed multidrug-resistant (MDR) [22,23].

## 3. Results

*Enterococcus* spp. from forty poultry farms were examined and identified. *Enterococcus faecalis* was isolated from 15 farms (37.5%), while *E. faecium* was detected in 17 (42.5%). *E. hirae* was identified in six poultry establishments (15%). One farm each (2.5%) harbored *E. durans* and *E. thialandicus*. Out of 40 single isolates per farm, 13 (32.5%) were found to be susceptible to antibiotics while 14 (35%) were multidrug-resistant.

All isolates from broiler breeder farms were identified as *E. faecium* and were resistant to at least two antibiotics or one or two classes, whereas it was found that there were no MDR isolates present (Table 1 and Table 2).

Among the eleven isolates from the laying hen farms, four were *E. faecalis* (36.36%), three *E. faecium* (27.27%), three *E. hirae* (27.27%), and one *E. durans* (9.09%). Susceptibility to all examined antimicrobial classes was established for six out of these eleven isolates (54.54%), while one *E. faecium* (9.09%) showed resistance to one class (tetracyclines) one *E. faecalis* (9.09%) to two classes, and three isolates (27.27%) were resistant to three or more classes of antimicrobials (Table 1 and Table 2). All MDR isolates were identified as *E. faecalis* (Table 1). Five isolates from 24 broiler farms (24%) were susceptible to all antimicrobials with two isolates identified as *E. faecalis* and another three as *E. faecium* (Table 1). Six isolates from broiler farms (25%)—four *E. faecalis*, one *E. faecium*, and one *E. hirae*—were resistant to two classes of antimicrobials. Eleven isolates from broiler farms were MDR, as presented in Table 1 and Table 2. Both isolates from the turkey farms were identified as *E. faecium* and both were susceptible to all tested antimicrobials (Table 1).

Fluoroquinolone resistance was verified in 15 isolates (37.5%), all of which also had an MDR status. Only one isolate (number 20) was sensitive to levofloxacin (LVX) even if resistance was detected to CIP, MXF, and NXN. Also, in one isolate from a broiler farm (number 31), resistance was detected to moxifloxacin (MXF), but not to CIP, NXN, or LVX. The most frequent resistance phenotype was to tetracycline, found in 24 out of 40 isolates (60%), followed by doxycycline resistance, detected in 21 isolates (52.5%). Resistance to erythromycin was confirmed in 13 isolates (32.5%) (Table 3). 

High-level resistance to streptomycin was present in 11 poultry isolates (27.5%). Resistance to quinupristin–dalfopristin (QDF) in *E. faecium* was found in only one isolate (No. 16) (2.5%) originating from a flock of broiler chickens. One isolate of *E. faecium* from a broiler farm (isolate number 16) was MDR to six antibiotic classes. None of the isolates were resistant to vancomycin or linezolid antibiotics.

## 4. Discussion

This work is the first attempt to determine the occurrence and resistotypes of *Enterococcus* species in environmental samples from poultry in Serbia. Unexpectedly, antimicrobial resistance was established in 27 isolates (67.5%), while isolates from 14 poultry farms (35%) were multidrug-resistant. Fluoroquinolone (FQ) resistance was also significant, being detected in *Enterococcus* spp. isolates from 15 poultry farms (37.5%). Such high-level resistance raises concerns about food safety in Serbia. In this study, the resistance against chemically improved fluoroquinolones, which exhibit better anti-bacillar activity against Gram-positive microorganisms, such as moxifloxacin, levofloxacin, and norfloxacin, is pinpointed. Newer drugs, descendants of ciprofloxacin, are important in human medicine due to their quick absorption and distribution to many tissues and body fluids. Therefore, they may be used, with caution, for the treatment of hospital- and community-acquired infections [24]. Fluoroquinolones inhibit bacterial DNA synthesis by acting on target enzymes or by activating multidrug exporters. Double mutations on *gyrA* and *parC* genes in *Streptococcus pneumoniae* induce high-level resistance to ciprofloxacin. The order of mutations depends on the type of FQ used for the therapy in some Gram-positive bacteria. Consequently, it is essential to carefully assess antibiotic resistance in indicator microorganisms such *Enterococcus* spp. Enrofloxacin (ENR) was removed from the chicken business in the United States in 2005 due to the serious health risks that resistance to ENR posed for humans infected with *Campylobacter* spp. [25,26]. Likewise, the administration of enrofloxacin in the poultry industry in Serbia has caused resistance to nalidixic acid in *Salmonella* Infantis isolates [27,28] and in avian pathogenic and commensal *Escherichia coli* as well [29], but also in *Campylobacter* isolates from human patients [30]. With this work, it has now been proven that resistance to FQ also occurs in Gram-positive bacteria from poultry origin such as *Enterococcus* spp. 

*Enterococcus* spp. have become important pathogens in veterinary medicine because of their resistance to vancomycin [31]. In poultry, vancomycin resistance is caused by the misuse of the growth promoter avoparcin, which belongs to glycopeptide antibiotics, chemically similar to vancomycin. However, in this research, resistance to vancomycin was not detected in the collection of isolates from poultry flocks. To the best of our knowledge, avoparcin was not used in Serbia as a feed additive, perhaps due its high price. Therefore, the absence of resistance to vancomycin in *Enterococcus* from poultry litter was not surprising. However, it is doubtful whether this absence is absolute: monitoring has not been established and there is a fair chance that isolates originating from ceca collected in slaughterhouses may provide different results and that vancomycin resistance will be found. 

The Serbian poultry industry depends on the import of breeding flocks and often also layer chickens. *Enterococci* from these birds may carry vancomycin-resistant genes. The risks of antibiotic therapy in food-producing animals must be carefully considered, taking into account the advantages and disadvantages, because new resistance mechanisms may emerge and some of the infectious clones become well established in different environments all around the globe. Before and after the ban of avoparcin in developed countries, some controversy has arisen regarding the role of medicinal food (a homogeneous mixture of feed and veterinary medicinal products, including antibiotics) in the spread of resistance to glycopeptide antibiotics; however, it was difficult to prove the epidemiological links between enterococci in food animals and isolates from human patients [32]. Similar controversy exists in evaluating the role of virginiamycin feed additive in the development of resistance to the streptogramin antibiotics quinupristin and dalfopristin in *E. faecium*. The transferable *vat* genes may support the spread of streptogramin-resistant isolates in farm animals, which could be transferred to humans via contaminated meat and environments. However, streptogramin-resistant *E. faecium* is well adapted to the cloaca and does not colonize the gut efficiently as it barely survives the gastric barrier [32]. 

Many isolates in this work were resistant to erythromycin and tetracycline. Such a resistance phenotype is a matter of concern in human medicine [33]. It could be assumed that antibiotic-resistant *Enterococcus* spp. from the environment can colonize poultry intestines and create a food contamination hazard during processing. The dissemination of such environmental bacteria through contaminated poultry litter is another important issue, especially in developing countries, where the disposal of bedding material from poultry houses is not safe [34]. Interestingly, only isolates from broilers and laying hens proved to be multidrug-resistant. This could be pure coincidence or due to the fact that the breeders and the two turkey flocks were not treated with antibiotics as frequently as broilers and layers. In our study, and in accordance with previous observations, we observed that the differential frequency of treatment among poultry types, such as broilers and turkeys, is influenced by various factors including the specific health challenges and management practices associated with each type. 

The genome plasticity of enterococci spp. is characterized by their ability to acquire new genes and genetic elements and the capacity to transfer large amounts of genetic material from one strain to another [13]. These properties have led to the formation of well-established clones in hospital and community settings [2]. It is noteworthy that some *E. faecalis* isolates from poultry and humans were found to be genetically indistinguishable in several independent studies [8]. It was also discovered that the *vanA* gene of the vancomycin-resistant *E. faecium* from poultry, ingested by healthy volunteers, can transfer resistance genes to human commensal *E. faecium*, causing transient colonization of the intestines [8]. Interestingly, genes encoding resistance to quinupristin/dalfopristin and to erythromycin were also transferable together with the *vanA* gene from resistant to susceptible *E. faecium* in the intestines of one out of six healthy volunteers [8,35]. Considering all of the above-mentioned facts, *Enterococcus* spp. are bacteria of great importance to public health, which require regular control of their resistotype and the study of transmission routes to both humans and animals. 

Owing to the possibility of acquiring resistance genes against several antibiotic classes, including glycopeptides, the significance of *Enterococcus* spp. in human and veterinary medicine has increased over the past few years [8]. These bacteria are intrinsically resistant to low doses of aminoglycosides (gentamicin and streptomycin) and cephalosporines. In addition, *E. faecalis* is intrinsically resistant to quinupristin/dalfopristin. Data from the European Antimicrobial Susceptibility Surveillance in Animals (EASSA) has shown that, except for erythromycin, tetracycline, and quinupristin/dalfopristin, clinical resistance to antibiotics was not found, or was rare in enterococci originating from cattle, pigs, and poultry. In 2014, a marked decrease in resistance to vancomycin in the EU was observed in comparison to previous reports from 2002 [36]. Similar results were obtained by Rebelo et al. [37]. Also, although resistance to linezolid is on the rise in nosocomial isolates, animals were not yet carriers of *Enterococcus* spp. with resistance to critical antibiotics [37]. Given that *Enterococcus* spp. are susceptible to transspecies transmission, antimicrobial resistance surveillance is of global importance. 

The main limitation of the present study is the relatively small number of samples. The number of samples in the present study was limited, but we ensured that the sampling methodology was representative of the region under study. The epizootiological area in our study encompasses two districts: South Backa and Srem. We made sure to include all representative farms from these districts to ensure comprehensive coverage of the region.

The inclusion of various types of poultry, including broiler breeders, laying hens, broiler chickens, and turkeys, was intentional to capture a broad spectrum of the poultry farming practices in the region. This diversity provides a more comprehensive understanding of the poultry industry. Regarding the inclusion of turkey farms, it is important to note that turkey farms are quite rare, not only in the specified districts, but also throughout the entire country. We recognize that only two turkey farms were included. However, this reflects the actual distribution and prevalence of turkey farms in the region, where broiler chicken farms are more predominant. The limited number of turkey farms in our sample accurately reflects their scarcity in the region. Thus, it is proportional to the local situation. Future studies will aim to increase the sample size and to cover a larger geographic area of the study and achieve an even more balanced representation of different poultry types. 

## 5. Conclusions

Because meat, meat products, and other foodstuffs can be contaminated with enterococci in addition to poultry litter and manure, it is important to continuously examine these bacteria of human, animal, and environmental origin. The systematic analysis of antimicrobial resistance in various *Enterococcus* spp. sampled from poultry farms in Serbia may provide insights into the epidemiology and determinants of resistance, which could help researchers to develop adequate strategies for antimicrobial stewardship and public health interventions. 

## Figures and Tables

**Table 1:** Resistotype of *Enterococcus* spp. isolates from poultry and turkey farms.

No.	Farm	Age of Birds	Resistotype	PCR/MALDI Identification	Resistance to Antibiotic Classes
1	Broiler breeder farm	21 weeks	TET, DOX	*E. faecium*	1
2	Broiler breeder farm	46 weeks	CIP, TET, DOX, MXF, NXN, LVX	*E. faecium*	2
3	Broiler breeder farm	60 weeks	TET, DOX	*E. faecium*	1
4	Laying hen farm	51 weeks	-	*E. faecium*	-
5	Laying hen farm	88 weeks	-	*E. faecium*	-
6	Laying hen farm	59 weeks	-	*E. hirae*	-
7	Laying hen farm	59 weeks	-	*E. hirae*	-
8	Laying hen farm	26 weeks	ERY, TET, DOX	*E. faecalis*	2
9	Laying hen farm	15 weeks	TET, DOX	*E. faecium*	1
10	Laying hen farm	120 weeks	CIP, TET, DOX, MXF, NXN, LVX, STR300	*E. faecalis*	3
11	Laying hen farm	135 weeks	-	*E. hirae*	-
12	Laying hen farm	14 weeks	ERY, TET, DOX, STR300	*E. faecalis*	3
13	Laying hen farm	15 weeks	ERY, TET, DOX, STR300	*E. faecalis*	3
14	Laying hen farm	26 weeks	-	*E. durans*	-
15	Broiler farm	4 weeks	CIP, TET, MXF, NXN, LVX	*E. faecium*	2
16	Broiler farm	4.5 weeks	CIP, ERY, CHL, TET, QDF, DOX, MXF, NXN, LVX, STR300	*E. faecium*	6
17	Broiler farm	5 weeks	CIP, ERY, TET, DOX, MXF, NXN, LVX, STR300	*E. faecalis*	4
18	Broiler farm	4 weeks	CIP, TET, DOX, MXF, NXN, LVX	*E. faecalis*	2
19	Broiler farm	4 weeks	CIP, FTN, MXF, NXN, LVX	*E. hirae*	2
20	Broiler farm	6 weeks	CIP, ERY, TET, FTN, DOX, MXF, NXN	*E. thialandicus*	4
21	Broiler farm	5 weeks	CIP, TET, FTN, DOX, MXF, NXN, LVX	*E. hirae*	3
22	Broiler farm	5 weeks	CIP, FTN, MXF, NXN, LVX, STR300	*E. hirae*	3
23	Broiler farm	4 weeks	-	*E. faecalis*	-
24	Broiler farm	5 weeks	-	*E. faecium*	-
25	Broiler farm	5 weeks	CIP, ERY, TET, DOX, MXF, NXN, LVX, STR300	*E. faecalis*	4
26	Broiler farm	4 weeks	ERY, TET, FTN, DOX	*E. faecium*	3
27	Broiler farm	2 weeks	CIP, ERY, TET, QDF, DOX, MXF, NXN, LVX	*E. faecalis*	4
28	Broiler farm	8 weeks	-	*E. faecalis*	-
29	Broiler farm	5 weeks	CIP, TET, DOX, MXF, NXN, LVX	*E. faecalis*	2
30	Broiler farm	6 weeks	TET, DOX	*E. faecium*	1
31	Broiler farm	6 weeks	ERY, TET, DOX, STR300, MXF	*E. faecium*	4
32	Broiler farm	5 weeks	ERY, STR300	*E. faecalis*	2
33	Broiler farm	5 weeks	TET, DOX	*E. faecalis*	1
34	Broiler farm	6 weeks	CIP, ERY, TET, DOX, MXF, NXN, LVX, STR300	*E. faecium*	4
35	Broiler farm	1 day	-	*E. faecium*	-
36	Broiler farm	4 weeks	-	*E. faecium*	-
37	Broiler farm	5 weeks	CIP, TET, MXF, NXN, LVX, STR300	*E. faecalis*	3
38	Broiler farm	4 weeks	ERY, TET	*E. faecalis*	2
39	Turkey farm	6 weeks	-	*E. faecium*	-
40	Turkey farm	16 weeks	-	*E. faecium*	-

Antibiotic disk abbreviation: ciprofloxacin (CIP), erythromycin (ERY), chloramphenicol (CHL), tetracycline (TET), nitrofurantoin (FTN), quinupristin–dalfopristin (QDF), doxycycline (DOX), moxifloxacin (MXF), norfloxacin (NXN), levofloxacin (LVX), streptomycin (STR300). -—Susceptible.

**Table 2:** Multidrug-resistant (MDR) *Enterococcus* spp. isolates from different types of farms.

Type of Farm	Number (percent) of MDR Isolates	Percent of Total Samples	MDR Species (%)
Broiler breeder farm	0/3	0/40	-
Laying hen farm	3/11 (27.27%)	3/40 (7.5%)	*E. faecalis* (100%)
Broiler farm	11/24 (45.83%)	11/40 (27.5%)	*E. faecalis* (36.36%)
			*E. faecium* (36.36%)
			*E. hirae* (18.18%)
			*E. thialandicus* (9.09%)
Turkey farm	0/2	0/40	-
**Total**	**14/40 (35%)**		

**Table 3:** Antibiotic resistance of *Enterococcus* spp. isolates.

Species (n; % of Isolates)	Type of Farm (Number of Resistant Isolates)	Antibiotic Resistance (Number of Isolates)										
		CIP	ERY	CHL	TET	FTN	QDF	DOX	MXF	NXN	LVX	STR
*E. faecium* (10; 25%)	Broiler breeder farm (3)	1			3			3	1	1	1	
	Laying hens farm (1)				1			1				
	Broiler farm (6)	3	4	1	6	1	1	5	4	3	3	3
*E. faecalis* (13; 32.5%)	Laying hens farm (4)	1	3		4			4	1	1	1	3
	Broiler farm (9)	6	5		8			6	6	6	6	4
*E. hirae* (3; 7.5%)	Broiler farm (3)	3			1	3		1	3	3	3	1
*E. thialandicus* (1; 2.5%)	Broiler farm (1)	1	1		1	1		1	1	1		
Total (27; 67.5%)		15	13	1	24	5	1	21	16	15	14	11

Antibiotic disks abbreviation: ciprofloxacin (CIP), erythromycin (ERY), chloramphenicol (CHL), tetracycline (TET), nitrofurantoin (FTN), quinupristin–dalfopristin (QDF),doxycycline (DOX), moxifloxacin (MXF), norfloxacin (NXN), levofloxacin (LVX), streptomycin (STR). None of the isolates were resistant to ampicillin (AMP), gentamicin (GMN), fosfomycin (FOS), linezolid (LZD), vancomycin (VAN), teicoplanin (TEC), or tigecycline (TGC).

## Data Availability

The original contributions presented in the study are included in the article, further inquiries can be directed to the corresponding author.

## References

[1] Shepard B.D., Gilmore M.S. (2002). Antibiotic-resistant enterococci: The mechanism and dynamics of drug introduction and resistance. Microbes Infect..

[2] Arias C.A., Murray B.E. (2012). The rise of the *Enterococcus*: Beyond vancomycin resistance. Nat. Rev. Microbiol..

[3] Krawczyk B., Wityk P., Gałęcka M., Michalik M. (2021). The many faces of *Enterococcus* spp.—Commensal, probiotic and opportunistic pathogen. Microorganisms.

[4] Osman K.M., Badr J., Orabi A., Elbehiry A., Saad A., Ibrahim M.D.S., Hanafi M.H. (2019). Poultry as a vector for emerging multidrug resistant *Enterococcus* spp: First report of vancomycin (van) and the chloramphenicol-florfenicol (cat-fex-cfr) resistance genes from pigeon and duck faeces. Microb. Pathog..

[5] Bortolaia V., Espinosa-Gongora C., Guardabassi L. (2016). Human health risks associated with antimicrobial-resistant enterococci and *Staphylococcus aureus* on poultry meat. Clin. Microbiol. Infect..

[6] Salam M.A., Al-Amin M.Y., Salam M.T., Pawar J.S., Akhter N., Rabaan A.A., Alqumber M.A.A. (2023). Antimicrobial Resistance: A Growing Serious Threat for Global Public Health. Healthcare.

[7] Nielsen S.S., Bicout D.J., Calistri P., Canali E., Drewe J.A., Garin-Bastuji B., Gonzales Rojas J.L., Gortazar Schmidt C., Herskin M., Michel V. (2021). Scientific Opinion on the assessment of animal diseases caused by bacteria resistant to antimicrobials: Poultry. EFSA J..

[8] Nielsen S.S., Bicout D.J., Calistri P., Canali E., Drewe J.A., Garin-Bastuji B., Gonzales Rojas J.L., Gortazar Schmidt C., Herskin M., Michel V. (2022). Scientific Opinion on the assessment of listing and categorisation of animal diseases within the framework of the Animal Health Law (Regulation (EU) No 2016/429): Antimicrobial-resistant *Enterococcus faecalis* in poultry. EFSA J..

[9] Van den Berghe E., De Winter T., De Vuyst L. (2006). Enterocin A production by *Enterococcus faecium* FAIR-E406 is characterized by a temperature-and pH-dependent switch-off mechanism when growth is limited due to nutrient depletion. Int. J. Food Microbiol..

[10] Dolka B., Chrobak-Chmiel D., Czopowicz M., Szeleszczuk P. (2017). Characterization of pathogenic *Enterococcus cecorum* from different poultry groups: Broiler chickens, layers, turkeys, and waterfowl. PLoS ONE.

[11] Nazeer N., Uribe-Diaz S., Rodriguez-Lecompte J.C., Ahmed M. (2021). Antimicrobial peptides as an alternative to relieve antimicrobial growth promoters in poultry. Br. Poult. Sci..

[12] Ribeiro J., Silva V., Monteiro A., Vieira-Pinto M., Igrejas G., Reis F.S., Barros L., Poeta P. (2023). Antibiotic resistance among gastrointestinal bacteria in broilers: A review focused on *Enterococcus* spp. and *Escherichia coli*. Animals.

[13] Chajecka-Wierzchowska W., Zadernowska A., Łaniewska-Trokenheim Ł. (2017). Virulence factors of *Enterococcus* spp. presented in food. LWT.

[14] Jung A., Rautenschlein S. (2014). Comprehensive report of an *Enterococcus cecorum* infection in a broiler flock in Northern Germany. BMC Vet. Res..

[15] Lake A., Fields R., Guerrero F., Almuzaini Y., Sundaresh K., Staffetti J. (2020). Case of *Enterococcus cecorum* human bacteremia, United States. Healthc. J. Med..

[16] Woo P.C.Y., Tam D.M.W., Lau S.K.P., Fung A.M.Y., Yuen K.Y. (2004). *Enterococcus cecorum* empyema thoracis successfully treated with cefotaxime. J. Clin. Microbiol..

[17] Petersen A., Chadfield M.S., Christensen J.P., Christensen H., Bisgaard M. (2008). Characterization of small-colony variants of *Enterococcus* faecalis isolated from chickens with amyloid arthropathy. J. Clin. Microbiol..

[18] (2018). Rulebook on Determining Measures for Early Detection, Diagnosis, Prevention of Spread, Control and Eradication of Poultry Infection with Certain Salmonella Serovars.

[19] Dutka-Malen S., Evers S., Courvalin P. (1995). Detection of glycopeptide resistance genotypes and identification to the species level of clinically relevant *Enterococci* by PCR. J. Clin. Microbiol..

[20] Clinical and Laboratory Standards Institute (CLSI) (2022). Performance Standards for Antimicrobial Susceptibility Testing, M100.

[21] European Committee on Antimicrobial Susceptibility Testing (EUCAST) (2022). Breakpoint Tables for Interpretation of MICs Zone Diameters.

[22] Schwarz S., Silley P., Simjee S., Woodford N., van Duijkeren E., Johnson A.P., Gaastra W. (2010). Editorial: Assessing the antimicrobial susceptibility of bacteria obtained from animals. J. Antimicrob. Chemother..

[23] Magiorakos A.P., Srinivasan A., Carey R.B., Carmeli Y., Falagas M.E., Giske C.G., Harbarth S., Hindler J.F., Kahlmeter G., Olsson-Liljequist B. (2012). Multidrug-resistant, extensively drug-resistant and pandrug-resistant bacteria: An international expert proposal for interim standard definitions for acquired resistance. Clin. Microbiol. Infect..

[24] Zhanel G.G., Walkty A., Vercaigne L., Karlowsky J.A., Embil J., Gin A.S., Hoban D.J. (1999). The new fluoroquinolones: A critical review. Can. J. Infect. Dis. Med. Microbiol..

[25] Colligon P. (2005). Fluoroquinolone use in food animals. Emerg. Infect. Dis..

[26] Nelson J.M., Chiller T.M., Powers J.H., Angulo F.J. (2007). Fluoroquinolone-resistant *Campylobacter* species and the withdrawal of fluoroquinolones from use in poultry: A public health success story. Clin. Infect. Dis..

[27] Jovčić B., Novović K., Filipić B., Velhner M., Todorović D., Matović K., Rašić Z., Nikolić S., Kiškarolj F., Kojić M. (2020). Genomic characteristics of colistin-resistant *Salmonella enterica* subsp. enterica serovar Infantis from poultry farms in the Republic of Serbia. Antibiotics.

[28] Velhner M., Kozoderović G., Grego E., Galić N., Stojanov I., Jelesić Z., Kehrenberg C. (2014). Clonal spread of *Salmonella enterica serovar* Infantis in Serbia: Acquisition of mutations in the topoisomerase genes gyrA and parC leads to increased resistance to fluoroquinolones. Zoononses Public Health.

[29] Velhner M., Todorović D., Grego E., Cerar Kišek T., Ljubojević D., Cvitković Špik V., Pajić M., Kozoderović G. (2020). Characterisation of multidrug resistant *Escherichia coli* from poultry litter and poultry carrying virulence genes for evaluation of poultry farm management practice. Eur. Poult. Sci..

[30] Bulajić N., Miljković-Selimović B., Tambur Z., Kocić B., Kalevski K., Aleksić E. (2022). Prevalence of antimicrobial resistance in *Campylobacter* spp.: A review of the literature. Acta Microbiol. Immunol. Hung..

[31] Van den Bogaard A.E., Stobberingh E.E. (1999). Contamination of animal feed by multiresistant enterococci. Lancet.

[32] Hao H., Sander P., Iqbal Z., Wang Y., Cheng G., Yuan Z. (2016). The risk of some veterinary antimicrobial agents on Public Health associated with antimicrobial resistance and their molecular basis. Front. Microbiol..

[33] Ahmadpoor N., Ahmadrajabi R., Esfahani S., Hojabri Z., Moshafi M.H., Saffari F. (2021). High-level resistance to erythromycin and tetracycline and dissemination of resistance determinants among clinical *Enteerococci* in Iran. Med. Princ. Pract..

[34] Ljubojević D., Puvača N., Pelić M., Todorović D., Pajić M., Milanov D., Velhner M. (2016). Epidemiological significance of poultry litter for spreading the antibiotic-resistant strains of *Escherichia coli*. Worlds Poult. Sci. J..

[35] Lester C.H., Frimodt-Møller N., Sørensen T.L., Monnet D.L., Hammerum A.M. (2006). In vivo transfer of the *vanA* resistance gene from an *Enterococcus faecium* isolate of animal origin to an *E. faecium* isolate of human origin in the intestines of human volunteers. Antimicrob. Agents Chemother..

[36] de Joung A., Simjee S., Rose M., Moyaert H., El Garch F., Youala M., on behalf of the EASSA study group (2019). Antimicrobial resistance monitoring in commensal enterococci from healthy cattle, pigs and chickens across Europe during 2004–14 (EASSA Study). J. Antimicrob. Chemother..

[37] Rebelo A., Duarte B., Ferreira C., Mourão J., Ribeiro S., Freitas A.R., Coque T.M., Willems R., Corander J., Piexel L. (2023). *Enterococcus* spp. from chicken meat collected 20 years apart overcome multiple stresses occurring in the poultry production chain: Antibiotics, copper and acids. Int. J. Food Microbiol..

